# Hormonal and metabolites responses in Fusarium wilt-susceptible and -resistant watermelon plants during plant-pathogen interactions

**DOI:** 10.1186/s12870-020-02686-9

**Published:** 2020-10-22

**Authors:** Deepak M. Kasote, G. K. Jayaprakasha, Kevin Ong, Kevin M. Crosby, Bhimanagouda S. Patil

**Affiliations:** 1 Vegetable and Fruit Improvement Center, Department of Horticultural Sciences, Texas A&M University, 1500 Research Parkway, Suite A120, College Station, TX 77845 USA; 2 Texas Plant Disease Diagnostic Laboratory, Texas A&M AgriLife Extension Service, College Station, TX 77843 USA

**Keywords:** *Fusarium oxysporum* f. sp. *niveum*, Indole-3-acetic acid, Jasmonic acid-isoleucine, Methyl jasmonate, Lysine, Melatonin, Metabolomics, Watermelon

## Abstract

**Background:**

*Fusarium oxysporum* f. sp. *niveum* (FON) causes Fusarium wilt in watermelon. Several disease-resistant watermelon varieties have been developed to combat Fusarium wilt. However, the key metabolites that mount defense responses in these watermelon varieties are unknown. Herein, we analyzed hormones, melatonin, phenolic acids, and amino acid profiles in the leaf tissue of FON zero (0)-resistant (PI-296341, Calhoun Grey, and Charleston Grey) and -susceptible (Sugar Baby) watermelon varieties before and after infection.

**Results:**

We found that jasmonic acid-isoleucine (JA-Ile) and methyl jasmonate (MeJA) were selectively accumulated in one or more studied resistant varieties upon infection. However, indole-3-acetic acid (IAA) was only observed in the FON 0 inoculated plants of all varieties on the 16th day of post-inoculation. The melatonin content of PI-296341 decreased upon infection. Conversely, melatonin was only detected in the FON 0 inoculated plants of Sugar Baby and Charleston Grey varieties. On the 16th day of post-inoculation, the lysine content in resistant varieties was significantly reduced, whereas it was found to be elevated in the susceptible variety.

**Conclusions:**

Taken together, Me-JA, JA-Ile, melatonin, and lysine may have crucial roles in developing defense responses against the FON 0 pathogen, and IAA can be a biomarker of FON 0 infection in watermelon plants.

## Background

Fusarium wilt in watermelon is caused by the fungus *Fusarium oxysporum* F. sp. *niveum* (FON). It is one of the major diseases of watermelon found in almost all production areas of tropical and subtropical regions of the world, including the USA [1, 2]. Several chemical, biological, and crop rotation methods, including grafting of watermelon scions onto FON-resistant rootstocks, have been usually advised to control fusarium wilt in watermelon [3]. However, the management of fusarium wilt is gradually becoming expensive, and even difficult due to the longer persistence of the FON pathogen in the soil, and the evolution of its new races [4]. In view of this, the use of disease-resistant varieties is considered one of the best methods for the management of fusarium wilt in watermelon.

Thus far, four FON races (0–3) have been identified in watermelon based on their aggressiveness or ability to overcome specific resistance in a set of differential watermelon cultivars [4]. Race 0 is the least aggressive race, mainly found to cause wilt on cultivars with no wilt-resistance genes, such as Sugar Baby [5]. Race 1 is a medium aggressive race, which causes slight to moderate wilt on most of the varieties that are classified as resistant to Fusarium wilt, for instance, Charleston Gray [5, 6]. The watermelon variety, Calhoun Grey was found to have strong resistance against FON-1 [6]. Race 2 is found to be highly aggressive, which can overcome the wilt-resistance in all commercial watermelon varieties [5]. Considering this, several accessions of wild watermelon were screened for resistance against Race 2 and found that two plant introductions (PIs) of USDA, PI 296341, and PI 271769 have resistance against Race 2 [7]. Single dominant genes, Fom-2, and Fom-1 conferred resistance against FON races 1 and 0, respectively [8]. In addition to race 2, a most virulent race 3 has been found in Florida, and Maryland, for which no watermelon resistant variety is commercially available [6, 9].

The FON infection in watermelon plants is a complex process, which consists of several stages of host-pathogen interactions [10]. The pathogen penetrates and colonizes watermelon roots by overcoming the barrier of plant defenses. In the final stages of infection, pathogen lytic enzymes, and toxins cause disease symptoms [10, 11]. Recently, transcriptomics and proteomics studies have been conducted to elucidate the molecular mechanism of FON pathogenicity, and watermelon plant immunity towards FON infection. A full-length enriched cDNA library was constructed from the root tissues of Fusarium wilt stressed watermelon variety PI296341, and demonstrated that the expression levels of genes of the transcription factor, ClWRKY1 reached a maximum level at 12 h after inoculation [12]. Moreover, the transcript levels of plant defensin-like genes ClPDF2.1 and ClPDF2.4, phenylalanine ammonia lyase, chitinase, and ascorbate peroxidase were significantly induced in the roots of a fusarium wilt-susceptible watermelon variety during FON 1 infection [10]. Antifungal thaumatin-like protein (ClTLP27) was identified in leaf tissue upon FON inoculation [13].

Besides genes and proteins, several metabolites of watermelon plants are also found to be involved in providing defense against FON infection. The FON-resistant variety was found to have a higher content of antifungal phenolic acids, such as 4-hydroxybenzoic acid, phthalic acid (PHA), and gallic acid (GAA) [14]. Similarly, a higher content of the free amino acids was reported in FON-resistant than FON-susceptible watermelon varieties [15]. However, there is little known information about the exact role of these metabolites in providing a defensive response at the different stages of FON-watermelon plant interactions.

Herein, to identify biomarkers of Fusarium wilt resistance in watermelon plants, we systematically analyzed the hormones, melatonin, phenolic acids, and amino acid levels in the leaf tissue of FON-resistant and -susceptible watermelon varieties after FON infection. In addition, an untargeted metabolomics approach was used to visualize metabolome changes in FON-resistant and susceptible watermelon varieties at different stages of plant-pathogen interactions.

## Results

### Metabolic profiling

**Fig. 1:**
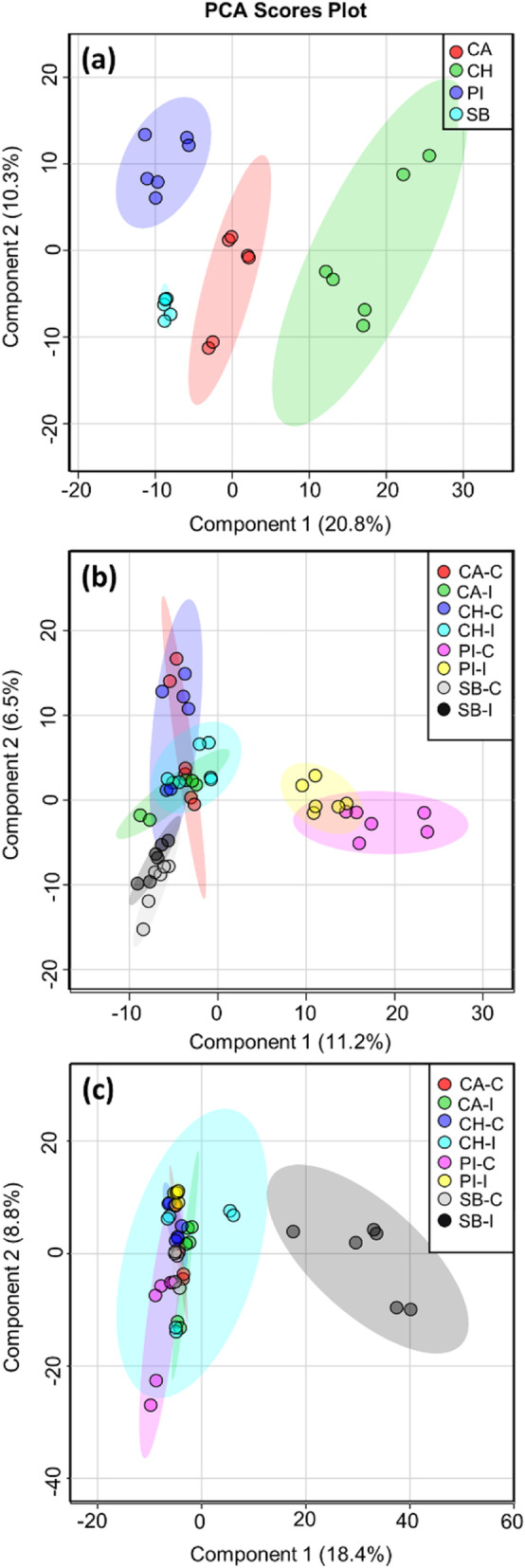
**a** Principal Component Analysis (PCA) scores plot of metabolome of watermelon varieties, PI-296341 (PI), Sugar Baby (SB), Calhoun Grey (CA) and Charleston Grey (CH) before *Fusarium oxysporum* F. sp. *niveum* 0 (FON 0) infection. **b** and **c** shows PCA scores plots of control (−C) and FON 0 inoculated (−I) plants metabolome of above differential watermelon varieties on the 8th and 16th day of post-infection, respectively

Figure 1 shows the principal component analysis (PCA) score plots of differential watermelon varieties before and after FON 0 infection (on the 8th and 16th day of post-inoculation). In Fig. 1a, the first two principal components (PCs) accounted for 31.1% of the total variance of the data and showed distinct clusters for each watermelon variety. On the 8th day of post-infection, both control and inoculated plants of each of the studied varieties showed some degree of overlapping, and the observed total variance of the data between the first two PCs was 17.7% (Fig. 1b). The control and inoculated group clusters of Calhoun Grey and Charleston Grey varieties were tightly grouped. They had some degree of overlapping with Sugar baby control and inoculated group clusters. Moreover, the control and an inoculated group of clusters for the PI-296341 were wholly segregated from the overlapped clusters of other variety groups (Fig. 1b). However, on the 16th day of post-infection, Sugar Baby FON 0-inoculated plants formed a discrete cluster from its respective control group plants, and also from untreated and inoculated a group of plants of other varieties (Figs. 1c, S2).

### Evaluation of hormonal levels at various stages of plant-pathogen interactions

**Fig. 2:**
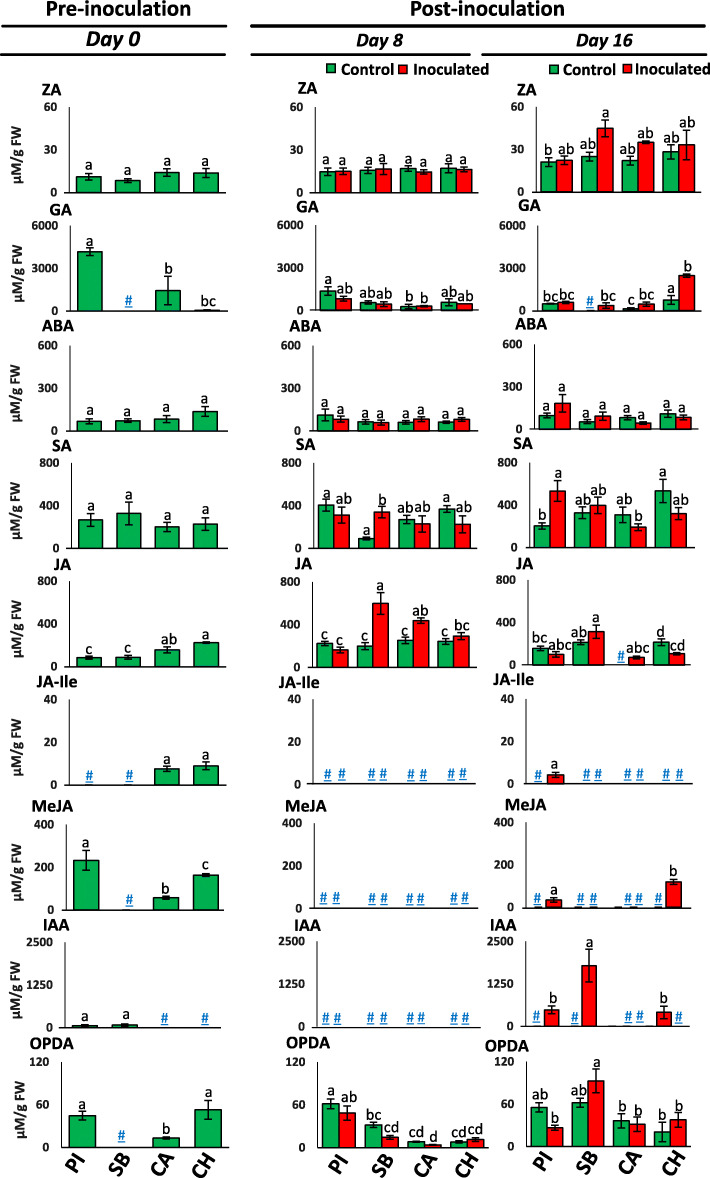
The hormones levels in the leaf tissue of FON 0 pre- and post-inoculated plants of watermelon varieties PI-296341 (PI), Sugar Baby (SB), Calhoun Grey (CA) and Charleston Grey (CH). Not detected level is indicated as hash-underscore (#). The significant differences (*P* < 0.05) among watermelon varieties are shown by different letters, based on a post hoc Tukey test. (ZA - zeatin, GA - gibberellic acid, ABA - abscisic acid, SA - salicylic acid, JA - jasmonic acid, JA-Ile - jasmonic acid-isoleucine, MeJA - methyl jasmonate, IAA - indole-3-acetic acid and OPDA - 12-oxo phytodienoic acid)

The leaf hormone levels in the differential watermelon varieties, PI-296341, Sugar Baby, Calhoun Grey, and Charleston Grey, at FON 0 pre- and a post-inoculated stages are shown in Fig. 2. At pre-inoculation stage (in 10 day old seedlings), all studied FON 0-resistant varieties such as PI-296341, Calhoun Grey, and Charleston Grey had detectable levels of gibberellic acid (GA), methyl jasmonate (MeJA), and 12-oxo-phytodienoic acid (OPDA). The leaf tissue of Sugar Baby showed the accumulation of GA and OPDA only in the pre-inoculation stage (8th and 16th day). The accumulation of MeJA was observed in the inoculated plants of PI-296341 and Charleston Grey in the late phase of the post-inoculation stage.

On the 8th day of post-inoculation, jasmonic acid (JA) and salicylic acid (SA) levels were significantly elevated in the FON 0 inoculated plants of Sugar Baby compared to its control plants. Similarly, FON 0 inoculated plants of Calhoun Grey also showed a significantly increased level of JA compared to the control plants on the 8th day of post-inoculation.

The levels of jasmonic acid-isoleucine (JA-Ile) was only detected at a pre-inoculation stage in the leaf tissues of Calhoun Grey and Charleston Grey seedlings. On the application of FON 0 pathogen, JA-Ile levels became non-detectable in all studied varieties and only appeared in the FON 0 inoculated plants of PI-296341 variety on the 16th day of post-inoculation. Similar to JA-Ile, hormone, indole-3-acetic acid (IAA) was predominantly observed in the FON 0 inoculated plants of PI-296341, Sugar Baby, and Charleston Grey varieties during the late phase (16th day) of post-inoculation.

### Melatonin content changes after FON infection

**Fig. 3:**
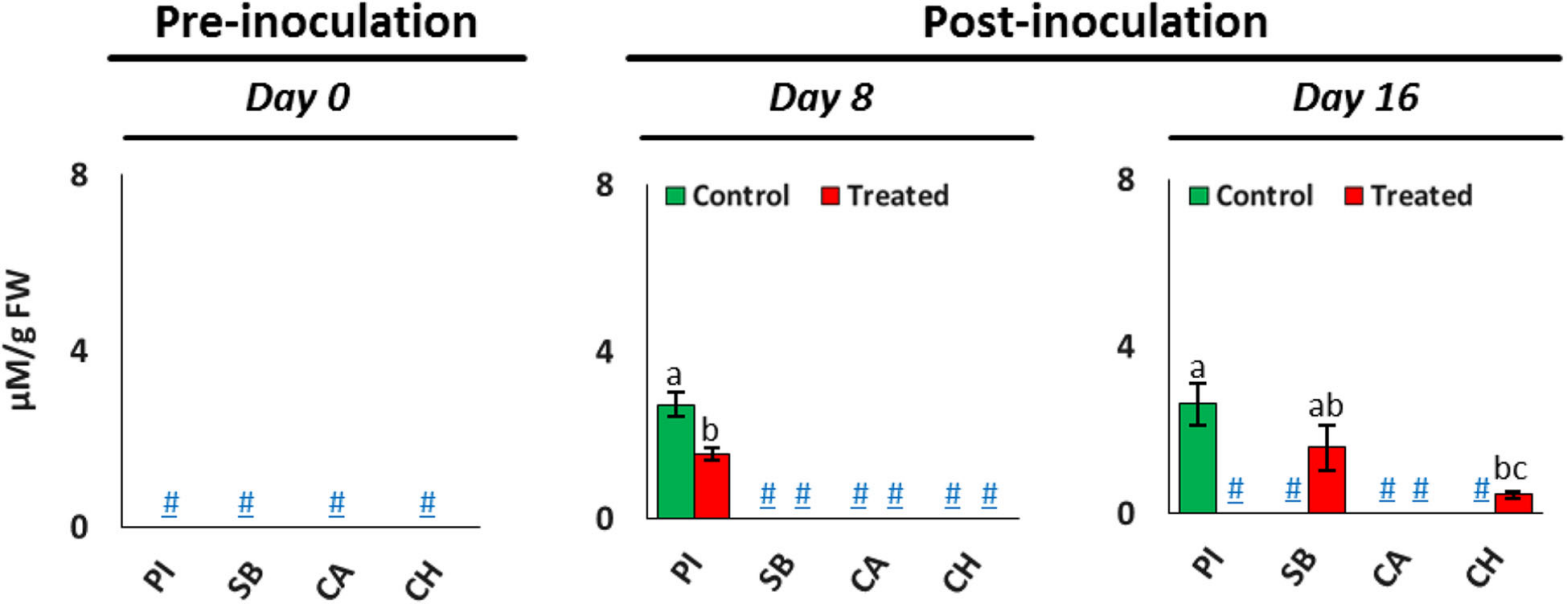
Melatonin content in the leaf tissue of FON 0 pre- and post-inoculated plants of watermelon varieties PI-296341 (PI), Sugar Baby (SB), Calhoun Grey (CA) and Charleston Grey (CH). Not detected level is indicated as hash-underscore (#). The significant differences (*P* < 0.05) among watermelon varieties are shown by different letters, based on a post hoc Tukey’s test

The results of quantitative profiles of melatonin content in the leaf tissues of differential watermelon varieties, PI-296341, Sugar Baby, Calhoun Grey, and Charleston Grey before and FON after the infection are depicted in Fig. 3. Interestingly, melatonin was not in the detectable range in the leaf tissues of all studied watermelon varieties at the pre-inoculation stage. After 8 days of infection, it was only detected in the control and group plants of PI-296341. Here, we found that melatonin content was significantly reduced in the leaf tissue of PI-296341 variety after infection. In addition, on the 16th day after infection, melatonin content was not detected in the leaf tissue of inoculated plants of watermelon varieties PI-296341 and Calhoun Grey. Conversely, it was only detected in the FON 0 inoculated plants of varieties Sugar Baby, and Charleston Grey.

### Free amino acids content in the differential watermelon varieties before and after FON infection

The levels of 10 different amino acids in the leaf tissues of PI-296341, Sugar Baby, Calhoun Grey, and Charleston Grey watermelon varieties before and after FON 0 infection are tabulated in Table 1. The leaf amino acid profiles of PI-296341, Sugar Baby, Calhoun Grey, and Charleston Grey varieties at the pre-inoculation stage were distinct. After 8 days of infection, lysine (Lys) content of leaf tissues of infected samples was significantly reduced in all studied varieties compared with their respective control samples. Interestingly, on the 16th day of infection, infected plants of resistant varieties (PI-296341, Calhoun Grey, and Charleston Grey) also had a reduced level of Lys. In contrary to this, leaf samples of infected sugar Baby plants showed a significantly elevated level of Lys, compared to its control plants (Table 1).

**Table 1:**
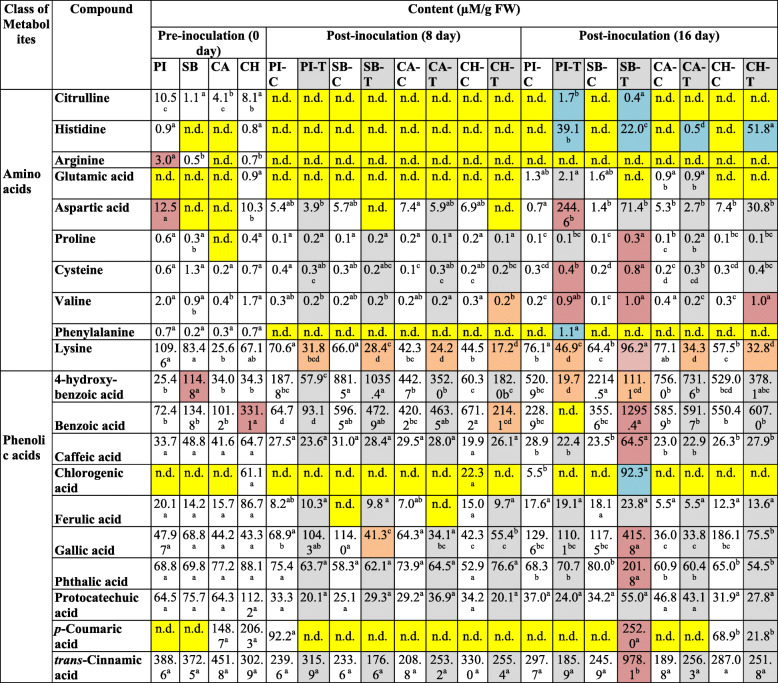
Changes in the amino acid and phenolic acid levels in pre- and post-inoculated plants of watermelon variety, PI-296341 (PI), Sugar Baby (SB), Calhoun Grey (CA) and Charleston Grey (CH). The letter ‘C’ and ‘T’ used denote control and treatment group plants, respectively. The significant differences (*P* < 0.05) among watermelon varieties are shown by different letters, based on a post hoc Tukey’s test. Different colors indicate not detected (yellow); significantly increased (pink-orange); significantly decreased (light green); only detected (light blue) levels

Similarly, the levels of some other amino acids were also altered on the 16th day of post FON 0 inoculation. The leaf tissue levels of valine and histidine were either in the detectable range or significantly elevated in infected plants of all studied varieties (Table 1). In addition, the level of proline was only found to be substantially increased in the infected Sugar Baby plants in the late stage of infection. This observation indicates over-accumulation of proline may be linked with the development of wilt symptoms.

### Phenolic acids levels in the differential watermelon varieties before and after FON infection

The results of leaf phenolic acids profiles of PI-296341, Sugar Baby, Calhoun Grey, and Charleston Grey varieties before and after FON 0 infection are shown in Table 1. Alteration in the phenolic acid levels was mainly observed at the late phase of post-inoculation (16th day), especially in the plants of susceptible variety, Sugar Baby. On the 16th day of infection, the contents of benzoic acid (BA), caffeic acid (CFA), GAA, and PHA were significantly increased in the leaf samples of infected plants of Sugar Baby, compared with its control plants. Similarly, chlorogenic acid (CGA) and *p*-coumaric acid (*p*CA) were characteristically detected in the leaf samples of infected plants of Sugar Baby.

## Discussion

### Untargeted metabolomics to visualize the state of FON infection and disease development in fusarium wilt-resistant and -susceptible watermelon varieties

In the pathogenicity test, the higher incidence of wilt was only observed on Sugar Baby among studied watermelon varieties. Except, Charleston Grey, other FON 0 resistant varieties such as PI-296341 and Calhoun Grey didn’t show any wilt symptoms up to 16th day of post-inoculation. Consequently, it was difficult to differentiate the pathogen infection and disease progression in the Fusarium wilt-resistant varieties. In recent studies, a green-fluorescent-protein-tagged isolate of FON 1 was used to determine the differences in infection, and to understand fungal-plant interactions in the root samples of Fusarium wilt-resistant and -susceptible varieties [10, 16]. In recent years, the metabolomics approach has been used to decipher the chemical defense in plants against pathogens [17]. In our study, untargeted metabolomics approach was used to visualize the state of FON infection and disease development in FON 0-resistant and susceptible watermelon varieties. The mathematical algorithm, PCA was used to reduce multidimensional UPLC/ESI-HR-QTOFMS data, and to generate a graphical output of plant-pathogen interaction in different Fusarium wilt-resistant and -susceptible varieties [18, 19]. The results of PCA analyses demonstrated that the metabolome of each studied watermelon variety was distinct (Fig. 1a), which indicates the probable diverse defense traits of these varieties against the studied pathogen. The observed slight segregation in PCA scores among the control and inoculated group clusters of PI-296341 and Sugar Baby varieties on the 8th day of post- infection may reflect the infection/colonization stage of the FON 0 pathogen in these plants (Fig. 1b). In addition, on the 16th day of post-infection, symptomatic and asymptomatic plants showed two distinct clusters on PCA score plots, irrespective of watermelon genotype (Fig. 1c). Moreover, we found that pathogen-inoculated plants of all studied resistant varieties formed a single cluster in PCA score plot compared the with susceptible variety, Sugar Baby (Figure S2), demonstrating resistant and susceptible watermelon varieties may have distinct metabolites responses against FON-0 pathogen in the later stage of infection. These findings suggest a potential role for the PCA technique in distinguishing the fusarium wilt symptomatic and asymptomatic plants. Similar to our study, PCA score plot has also been previously used to show the separations between the metabolic profiles of Powdery mildew susceptible and resistant rootstocks of watermelon [20]. After initial understanding about genotype-based metabolome changes in response to infection in watermelon plants, further targeted metabolomics studies were performed to identify biomarkers of fungal-plant interaction, and Fusarium wilt resistance in watermelon plants.

### Jasmonates (MeJA and JA-Ile) induced systemic resistance against FON 0, and IAA is a biomarker of fusarium wilt in watermelon

Plant hormones determine colonization and disease symptom development during plant-pathogen interactions [21]. In general, to defend themselves from pathogens, plants are dependent on intricate signaling networks regulated by phytohormones. Plant pathogens manipulate these hormone-regulated host defenses [22]. However, the overall role of plant hormones in plant-pathogen interactions is highly complex, and varies with plant and fungal genotypes [21]. Present results demonstrated that the FON 0 rewires hormone signaling networks distinctly in Fusarium wilt-susceptible and -resistant watermelon genotypes. The inoculated plants of Sugar Baby (susceptible variety) showed increased accumulation of major plant defense hormones SA and JA in the early phase of 0 infection (Fig. 2). Conversely, Fusarium wilt-resistant variety, Calhoun Grey only elevated the production of JA upon infection. This indicates that the induction of both JA and SA biosynthesis may occurs with the FON 0 colonization during the early episode of infection. It has been found that wheat intercropping increases the accumulation of SA in watermelon, and this induced SA enhances watermelon resistance to FON [2]. In the present study, an increase in SA was only observed in the leaf tissue of PI-296341 at the late phase of FON infection, which demonstrates that SA may contribute in providing added immunity against the FON 0 pathogen.

Jasmonates either promote resistance or susceptibility in FON-host plant interactions [21]. Here, we observed that Me-JA and JA-Ile accumulated in the resistant varieties in the late phase of infection (Fig. 2). This indicates that the accumulation of Me-JA and JA-Ile in the post-infection stage may be crucial in providing resistance against the FON 0 pathogen in watermelon. In the recent studies, exogenous MeJA treatment was found to induce disease resistance response in banana and wheat plants by increasing activities of defense enzymes and lowering reactive oxygen species (ROS) [23, 24]. Similarly, a boost in endogenous JA-Ile level is found to enhance the accumulation of phytoalexins in rice, and thereby, develop resistance against *Xanthomonas oryzae* pv. *oryzae* [25].

IAA is a key auxin produced by both plants and microbes, including *Fusarium* species [26]. Results of our study showed that IAA mainly accumulated in leaf tissue of FON 0 inoculated susceptible and resistant varieties in the late phase of infection (Fig. 2). This indicates that accumulated IAA may be fully or partly of fungal origin, as observed in *Fusarium graminearum* infection in wheat, and its endogenous concentration may be a decisive virulence factor in fusarium wilt symptoms development [27].

### Differential roles of melatonin in Fusarium wilt-susceptible and -resistant watermelon varieties

Melatonin is a ubiquitous molecule, which functions as an antioxidant or growth promoter in plants [28]. Recent studies confirmed that melatonin has a considerable role in developing resistance against Fusarium pathogens in different plants [29, 30]. It has been found that pathogen infection in plants triggers an oxidative burst, which subsequently induces endogenous biosynthesis of melatonin. This elevated melatonin level influences plant innate immunity through the mitogen-activated protein kinase (MAPK) pathway [31]. The catabolic products of melatonin such as 2-hydroxymelatonin (2HOM), cyclic-3-hydroxymelatonin (C3HOM), and N^1^-acetyl-N^2^-formyl-5-methoxyknuramine (AFMK) found to have higher ROS scavenging activity and also activate MAPK cascades as melatonin [31, 32]. Thus, by considering this, it has been proposed that increased melatonin in specialty crops such as watermelon is crucial to induce resistance against diverse filamentous pathogens [30]. Herein, we found that Fusarium wilt-resistant, PI-296341 had high melatonin content, which was significantly reduced upon FON 0 infection (Fig. 3). On the contrary, fusarium wilt-susceptible variety, Sugar Baby only accumulated melatonin in the late phase of FON infection, as an eventual weapon of defense. Taken together, present results showed that high endogenous melatonin content, including the formation of more bioactive metabolites from melatonin such as 2HOM, C3HOM, and AFMK may be essential in developing resistance against the FON 0 pathogen in watermelon plants.

### Catabolism of Lys may intensify watermelon plant defense against FON 0 pathogen

**Fig. 4:**
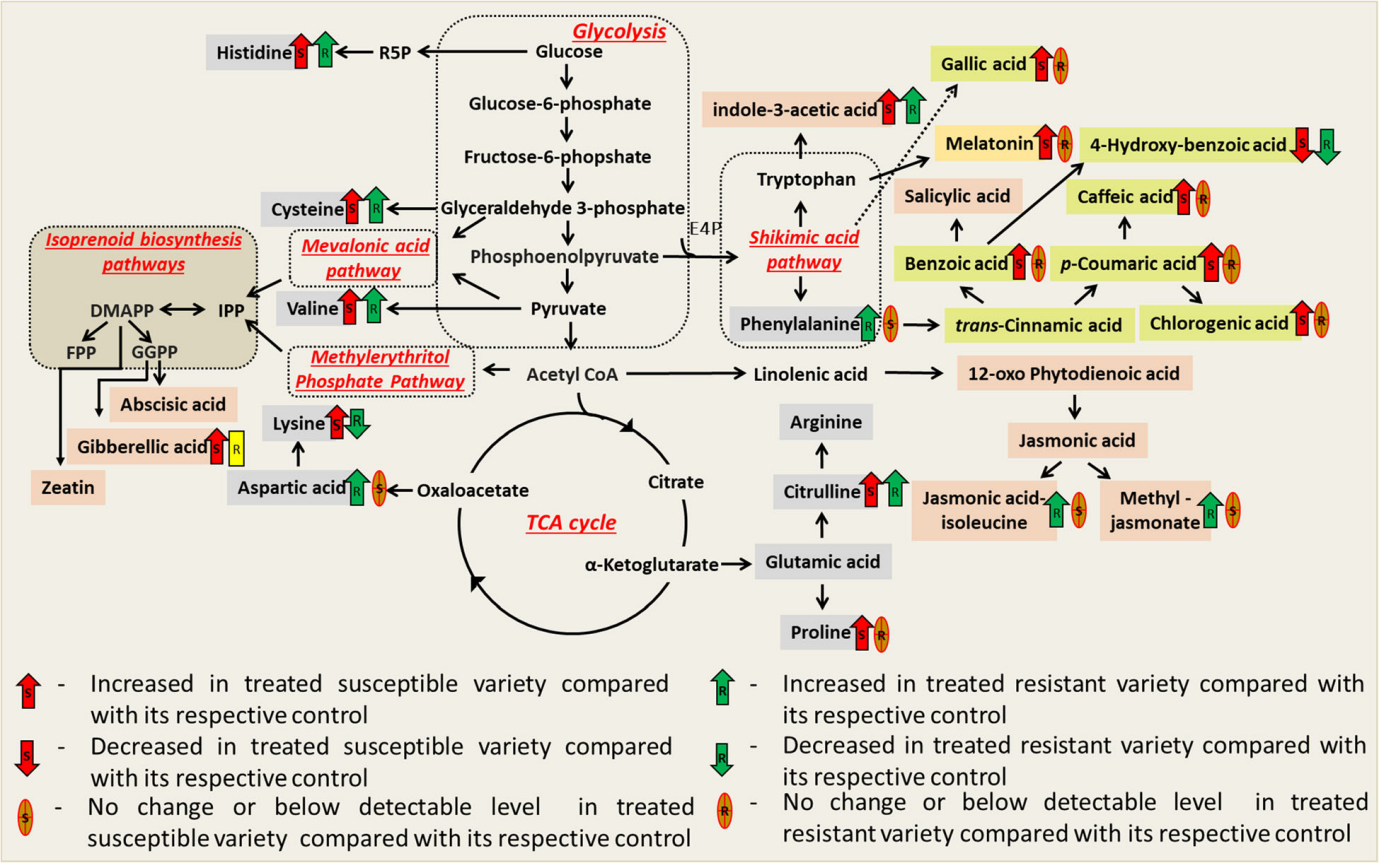
The metabolic pathway network signature of analyzed hormones and metabolites in the leaf tissues of Fusarium wilt-susceptible and -resistant plants of differential watermelon varieties on the 16th day after *Fusarium oxysporum* F. sp. *niveum* (FON 0) infection. Hormones, melatonin, amino acids, and phenolic acids are shown faint organ, mustard, grey, and pineapple colored text boxes. The red and green color arrows indicate changes in metabolites in susceptible and resistant watermelon varieties, respectively. Up arrowheads denote increased levels, whereas down arrowheads show decreased levels. Crossed oval shapes indicate no change or below detectable range. Abbreviations: R5P, ribose 5-phosphate; IPP, isopentenyl pyrophosphate; DMAPP, dimethylallyl pyrophosphate; FPP, farnesyl pyrophosphate; GGPP, geranylgeranyl pyrophosphate [35–37]

In plants, the metabolism of amino acids is the key event in the biosynthesis of several downstream natural products such as lignin and defense compounds [33]. Results of the present study showed that anabolism of most of the studied free amino acids increased at the late phase of post-inoculation in both fusarium wilt-susceptible and -resistant varieties (Table 1, Fig. 4); this indicates that free amino acids, predominantly histidine, citrulline, valine, and phenylalanine are involved in watermelon plant defense response. Interestingly, the accumulation of lysine was found to be decreased at post-inoculation, especially in Fusarium wilt-resistant varieties. This finding highlights that the increased catabolism of lysine is a crucial event in inducing defense against FON 0 pathogen. Recently, it has been found that the catabolism of lysine produces immune inducer pipecolic acid [34]. The pipecolic acid is an acyclic, non-protein amino acid, which was found to accumulate in the xylem sap and leaves of soybean seedlings following *Fusarium virguliforme* infection [34, 35]. Based on these literature reports and our observations, watermelon plants may accumulate pipecolic acid in the leaf tissues to defend from the FON 0 pathogen by increasing catabolism of lysine.

### Phenolic acids signature may characterize Fusarium wilt symptomatic stage in watermelon

**Fig. 5:**
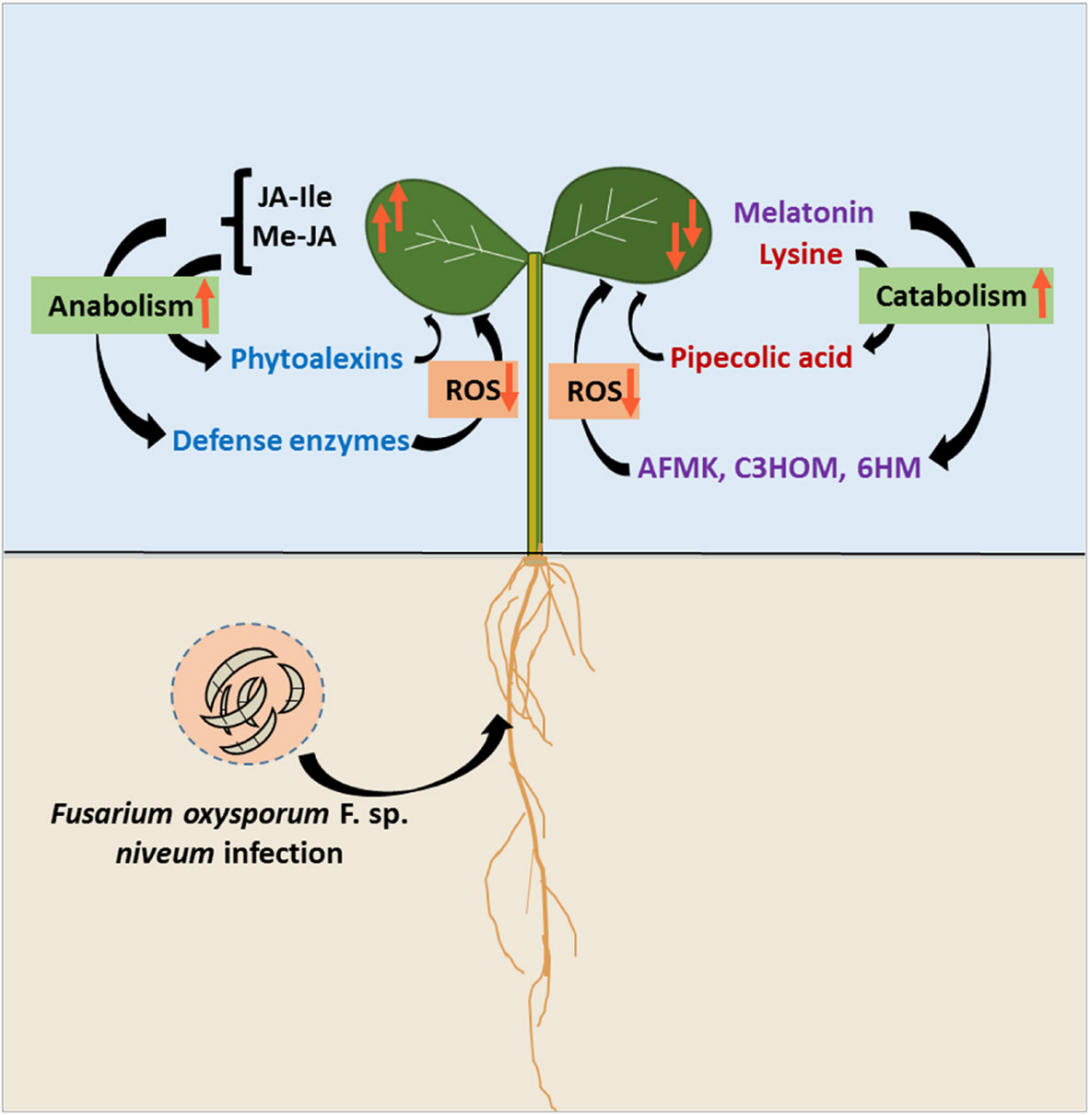
Pictorial representation of a presumptive multifaceted model of disease resistance mechanisms in Fusarium wilt-resistant watermelon varieties against *Fusarium oxysporum* F. sp. *niveum* (FON) pathogen. In Fusarium wilt-resistant watermelon varieties, Me-JA and JA-Ile accumulated upon infection and may provide induced resistance by the increased biosynthesis of defense enzymes and phytoalexins, including lowering of ROS. Conversely, high endogenous melatonin content of resistant watermelon varieties and/or formation of its catabolized metabolites such as 2-hydroxymelatonin (2HOM), cyclic-3-hydroxymelatonin (C3HOM), and N^1^-acetyl-N^2^-formyl-5-methoxyknuramine (AFMK) may develop added resistance in resistant varieties upon FON 0 infection. Similarly, pipecilic acid, a catabolic product of lysine, may further boost induced resistance in watermelon varieties

Phenolic acids are universally present in vascular plants and found to accumulate distinctly in compatible and incompatible interactions [36]. However, little is known about the exact role of phenolic acids signatures in plant defense and disease susceptibility. Our results showed that phenolic acids signatures of watermelon plants remain consistent during the early stage of FON 0 infection in both susceptible and resistant varieties. On the 16th day post-inoculation, the accumulation of most of the studied phenolic acids, such as BA, CFA, CGA, GAA, PHA, PCA, and TCA, significantly increased in leaf samples of the Fusarium wilt-susceptible variety, Sugar Baby (Table 1, Fig. 5). The *p*CA reported to have FON spore germination and reproduction inhibitory potential, and in contrast, ferulic acid, 4-hydroxybenzoic acid, and PHA found to promote FON spore germination and reproduction [37]. Since both FON inhibitory and FON promoting phenolic acids accumulated in the symptomatic stage of fusarium wilt, the ratio of FON inhibitory to promoting phenolic acids could be crucial for the progression of wilt symptoms.

## Conclusions

Herein, we have demonstrated that Me-JA, JA-Ile, melatonin, and Lys have a remarkable role in developing disease resistance responses in watermelon plants against the FON-0 pathogen. Based on our findings and other literature reports [19, 24, 25, 32, 34], we propose a multifaceted model of disease resistance in the watermelon plants gainst the FON 0 pathogen (Fig. 5), in which (i) Me-JA and JA-Ile accumulated in the resistant varieties at the post-infection stage, and may provide induced resistance by increasing biosynthesis of defense enzymes and phytoalexins, including lowering of ROS, as reported in wheat agints *Fusarium culmorum* [24]; and (ii) high endogenous melatonin content, and/or the formation of more bioactive metabolites from melatonin and lysine upon infection may further induced resistance in certain FON resistant watermelon varieties [31, 34]. Besides these biomarkers for disease resistance, we also identified IAA as a biomarker of FON-infection in certain watermelon genotypes, which can be used to monitor FON infection in watermelon plants. The overall aim of this was to identify metabolites involved in developing disease resistance in watermelon genotypes with differential FON resistance. Further multi-omics and correlation studies are warranted to decipher the differential disease resistance mechanisms in various watermelon FON resistant genotypes against different races of FON.

## Methods

### Chemicals reagents

Authentic standards of melatonin, phenolic acids (4-hydroxy-benzoic acid, benzoic acid, caffeic acid, chlorogenic acid, ferulic acid, gallic acid, protocatechuic acid, phthalic acid, *p*-coumaric acid, and trans-cinnamic acid), phytohormones (abscisic acid, gibberellic acid, jasmonic acid, methyl jasmonate, indole-3-acetic acid, salicylic acid, and zeatin), and amino acids (alanine, arginine, aspartic acid, asparagine, histidine, citrulline, cysteine, glutamic acid, lysine, phenylanine, proline, serine, threonine, tryptophan and valine) were procured from Sigma-Aldrich, St. Louis, MO, USA. Jasmonic acid-isoleucine and 12-oxo phytodienoic acid were procured from Cayman Chemical, MI, USA. All other chemicals used were of analytical grade.

### *Fusarium oxysporum* F. sp. *niveum* (FON) isolate, and inoculum preparation

The watermelon plants with typical fusarium wilt symptoms were collected from the commercial field of Premont, TX, USA in August 2017. For isolation of the pathogen, root and stem tissues were washed under running water. After surface sterilization and rewashing with water, pieces of these plant parts were placed on potato dextrose agar (PDA) plates, and incubated for 7–10 days at 25 °C. The isolate of pure culture was obtained by single spore culture technique [38]. The plates of 2 week old cultures of the FON isolate were used to prepare the inoculum. A conidial and mycelial fragment suspension was obtained by adding sterile saline on the culture plates and suspending the colonies in saline by gently scrubbing with a sterile plastic cell spreader.

### Plant materials, and seedling development

The seeds of Sugar Baby and Charleston Gray were purchased from Johnny’s Selected Seeds, Maine, USA. Calhoun Grey and PI-296341 seeds were received as samples from the USDA-ARS-GRIN repository in Griffin, GA, USA. Seeds of these four varieties were soaked in distilled water for 12 h. On the next day, after drying, 20 seeds of each variety in triplicate were sown in a plug tray (200 square cells) filled with potting soil (Metro-mix 900, Sun Gro Horticulture, Vancouver, Canada), and placed in a growth chamber (PGC Flex, Conviron, ND, USA) at 70% relative humidity under a 14 h/10 h (light/dark, 28 °C/18 °C) photo-period for germination and seedling development. On the 10th day, leaf samples of 3–4 seedlings of each variety were collected and stored at − 80 °C for metabolomics studies.

### Pathogenicity test, and race determination

Ten day old growth chamber grown seedlings samples from each variety were transplanted into 2.5″ square form pots containing potting soil. Seedlings from each variety were equally categorized into control and treatment groups. Subsequently, the seedlings from the treatment group were infected by placing 1 mL conidial and mycelial fragment suspension (2.6 × 10^5^ CFU) on their crowns using the sterile tip. Both inoculated and non-inoculated control plants were then transferred and grown in a greenhouse by following Biosafety Level 3 (BSL-3) guidelines. Disease symptoms were monitored every day for 16 days, and percentage of seedling showing symptoms of fusarium wilt were recorded (Average day/night greenhouse temperatures during the study were 31.1/22.2 °C). The criteria described by [6] was used to assign the race of FON, and to categorize studied watermelon varieties as susceptible (≥33% wilt) and resistant (< 33% wilt). Amongst studied differential watermelon varieties, Sugar Baby (susceptible to Race 0) was only showing a wilting incidence of over 33%. The FON isolate produced a minimal incidence of wilt on Charleston Grey (≥17) and had no disease incidence on PI-296341 and Calhoun Grey (Figure S1). Based on these observations, Premont, TX FON isolate (W-2-2) was confirmed as FON-zero (0) [16, 39]. For the metabolomics study, 3–4 seedling samples from each control and treated group were harvested on the 8th and 16th day of post-inoculation, and leaf samples were stored at − 80 °C until use.

### Quantification of melatonin, phenolic acids, and untargeted metabolomics analysis

The quantification of melatonin and phenolic acids, including untargeted metabolomics were performed using as per our previously described procedures [40]. Briefly, Frozen leaf tissue samples were ground in liquid nitrogen using a pestle, and 1 mL methanol was added to 50 mg of each sample. After vortexing and sonication, the mixture centrifuged (10,621 x g, 10 min). The separated supernatant was injected into a UPLC/ESI-HR-QTOFMS equipped with Eclipse Plus C18 RRHD (1.8 μm, 50 × 2.1 mm) column. The chromatographic separation and the mass spectrometer conditions were used, as described in our previous publications [19, 40, 41]. For the untargeted metabolomics study, similar data was further pre-processed by DataAnalysis Software, and information containing compounds specific mass, retention time, and peak intensity were extracted in Excel, and later exported to online software MetaboAnalyst 3.0 (http://www.metaboanalyst.ca/) for data normalization and multivariate analysis [42].

### Hormone analysis

The phytohormones analysis was carried out as per our earlier described procedure [19, 40]. The phytohormones were extracted by adding 1 mL extraction solvent, 2-propanol: water: acetic acid (80:19:1, v/v) to microcentrifuge tubes containing 50 mg pulverized leaf tissue in liquid nitrogen. Later on, The mixture was vortexed (30 s), sonicated, and centrifuged (10,621 x g for 10 min). The supernatant was separated and used for UPLC/ESI-HR-QTOFMS based quantitative profiling of hormones. The separation of hormones was performed on Eclipse Plus C18 RRHD (1.8 μm, 50 × 2.1 mm) column. The gradient binary mobile phase, 0.1% aqueous formic acid (A) and 0.1% formic acid in acetonitrile (B) with gradient program: 0 min, 0% B; 11 min, 80% B; 15 min, 100% B; 16 min 0% B, was used to conduct analysis. The column temperature was kept constant at 30 °C. The ESI(+)-MS/MS spectra were acquired at above mentioned mass spectrometer operating parameters [40].

### Amino acid profiling

The free amino acids from leaf tissue samples were extracted in 70% methanol, and quantitatively estimated using HPLC after dansyl-chloride (DNS-CL) derivatization, as per the procedure described earlier [19].

### Statistical analysis

Results were expressed as a mean ± standard error (SE) of two technical measurements of three biological replicates. MS Excel was used for regression analysis and data visualization. Statistical significance (*P* < 0.05) between treatment groups was assessed using the SPSS software (IBM SPSS Statistics, IBM Corp., Chicago, IL, USA).

## Supplementary information

**Additional file 1: Figure S1.** The pathogenicity test of Premont, TX *Fusarium oxysporum* F. sp. *niveum* (FON) isolate on differential watermelon varieties such as PI-296341, Sugar Baby, Calhoun Grey and Charleston Grey. Based on results of percentage of seedlings showing symptoms of fusarium wilt, Sugar Baby was identified as susceptible variety, and remaining three varieties such as PI-296341, Calhoun Grey and Charleston Grey confirmed as resistant to FON-0.

**Additional file 2: Figure S2.** (a) control and (b) *Fusarium oxysporum* F. sp. *niveum* 0 (FON 0) inoculated Principal Component Analysis (PCA) scores plot of watermelon varieties, PI-296341 (PI), Sugar Baby (SB), Calhoun Grey (CA) and Charleston Grey (CH) on the 16th day of post-infection.

## Data Availability

Datasets used in the current study are available from the corresponding author on reasonable request.

## Abbreviations

ABA: Abscisic acid
BA: Benzoic acid (BA)
C: Control
CA: Calhoun Grey
CFA: Caffeic acid
CH: Charleston Grey
DMAPP: Dimethylallyl pyrophosphate
FON: *Fusarium oxysporum* f. sp. *niveum*
FPP: Farnesyl pyrophosphate
GA: Gibberellic acid
GAA: Gallic acid
GGPP: Geranylgeranyl pyrophosphate
IAA: Indole-3-acetic acid
IPP: Isopentenyl pyrophosphate
JA: Jasmonic acid
JA-Ile: Jasmonic acid-isoleucine
Lys: Lysine
MeJA: Methyl jasmonate
OPDA: 12-oxo phytodienoic acid
OPDA: 12-oxo-phytodienoic acid
PCA: Principal component analysis
PCs: Principal components
PHA: Phthalic acid
PI: PI-296341
R5P: Ribose 5-phosphate
SA: Salicylic acid
SB: Sugar Baby
T: Treatment
ZA: Zeatin
