# Supplementary material for: Hormonal and metabolites responses in Fusarium wilt-susceptible and -resistant watermelon plants during plant-pathogen interactions
Source: BMC Plant Biol. 2020 Oct 22;20:481. doi: 10.1186/s12870-020-02686-9 (PMC7579875; doi:10.1186/s12870-020-02686-9)
Supplement: Supplementary file 1 — Additional file 1: Figure S1. The pathogenicity test of Premont, TX Fusarium oxysporum F. sp. niveum (FON) isolate on differential watermelon varieties such as PI-296341, Sugar Baby, Calhoun Grey and Charleston Grey. Based on results of percentage of seedlings showing symptoms of fusarium wilt, Sugar Baby was identified as susceptible variety, and remaining three varieties such as PI-296341, Calhoun Grey and Charleston Grey confirmed as resistant to FON-0. [file 12870_2020_2686_MOESM1_ESM.docx]

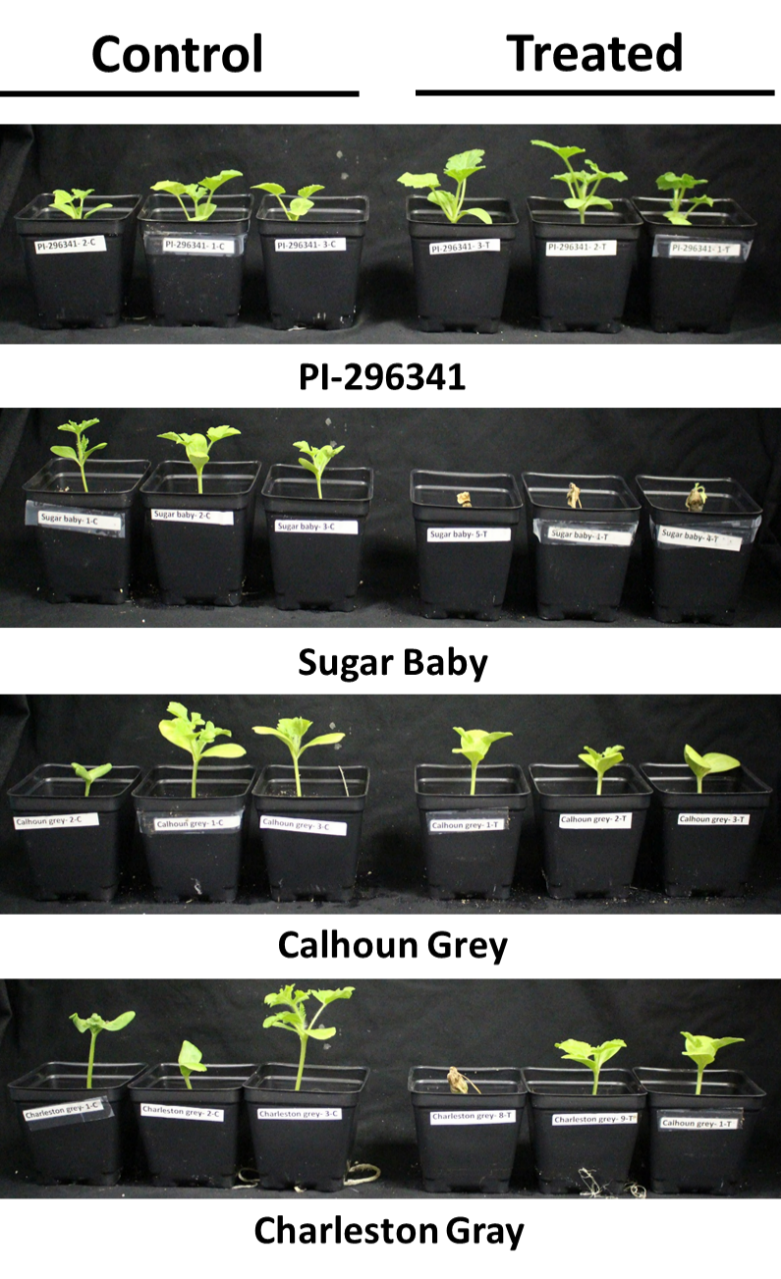


**Figure S1.** The pathogenicity test of Premont, TX *Fusarium oxysporum* F. sp. *niveum* (FON) isolate on differential watermelon varieties such as PI-296341, Sugar Baby, Calhoun Grey and Charleston Grey. Based on results of percentage of seedlings showing symptoms of fusarium wilt, Sugar Baby was identified as susceptible variety, and remaining three varieties such as PI-296341, Calhoun Grey and Charleston Grey confirmed as resistant to FON-0.
