# Supplementary material for: Hormonal and metabolites responses in Fusarium wilt-susceptible and -resistant watermelon plants during plant-pathogen interactions
Source: BMC Plant Biol. 2020 Oct 22;20:481. doi: 10.1186/s12870-020-02686-9 (PMC7579875; doi:10.1186/s12870-020-02686-9)
Supplement: Supplementary file 2 — Additional file 2: Figure S2. (a) control and (b) Fusarium oxysporum F. sp. niveum 0 (FON 0) inoculated Principal Component Analysis (PCA) scores plot of watermelon varieties, PI-296341 (PI), Sugar Baby (SB), Calhoun Grey (CA) and Charleston Grey (CH) on the 16th day of post-infection. [file 12870_2020_2686_MOESM2_ESM.docx]

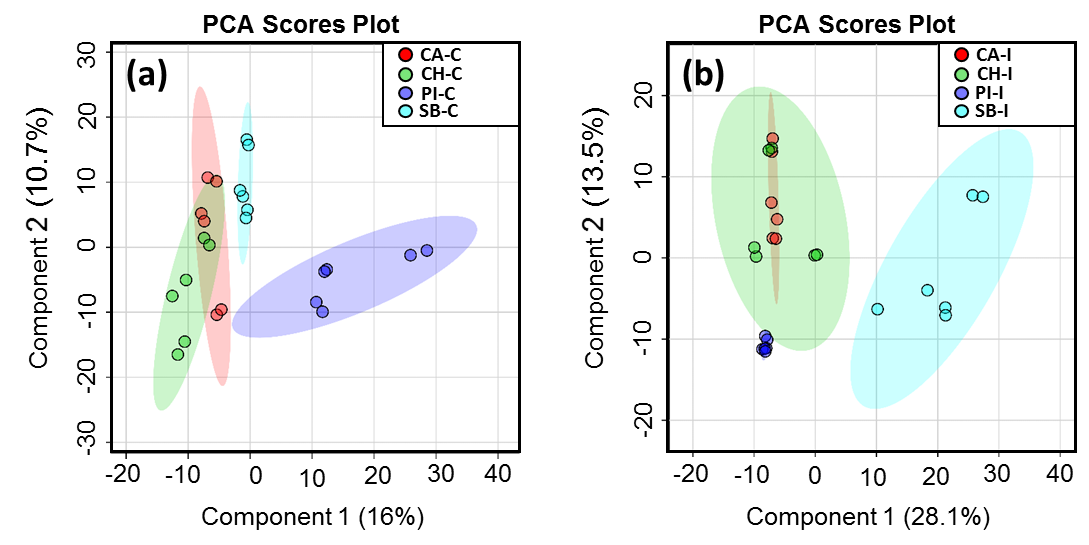


**Figure S2.** (a) control and (b) *Fusarium oxysporum* F. sp. *niveum* 0 (FON 0) inoculated principal Component Analysis (PCA) scores plot of watermelon varieties, PI-296341 (PI), Sugar Baby (SB), Calhoun Grey (CA) and Charleston Grey (CH) on the 16^th^ day of post-infection.
